# Pre-Slaughter Sources of Fresh Meat Quality Variation: The Case of Heavy Pigs Intended for Protected Designation of Origin Products

**DOI:** 10.3390/ani10122386

**Published:** 2020-12-14

**Authors:** Luca Sardi, Alessandro Gastaldo, Marzia Borciani, Andrea Bertolini, Valeria Musi, Anna Garavaldi, Giovanna Martelli, Damiano Cavallini, Eleonora Nannoni

**Affiliations:** 1 Department of Veterinary Medical Sciences, University of Bologna, Via Tolara di Sopra 50, 40064 Ozzano Emilia (BO), Italy; luca.sardi@unibo.it (L.S.); damiano.cavallini@unibo.it (D.C.); eleonora.nannoni2@unibo.it (E.N.); 2 Foundation C.R.P.A. (Research Centre on Animal Production) Studies and Researches, Viale Timavo 43/2, 42121 Reggio Emilia, Italy; a.gastaldo@crpa.it (A.G.); a.bertolini@crpa.it (A.B.); 3 C.R.P.A. (Research Centre on Animal Production), Viale Timavo 43/2, 42121 Reggio Emilia, Italy; m.borciani@crpa.it (M.B.); v.musi@crpa.it (V.M.); a.garavaldi@crpa.it (A.G.)

**Keywords:** animal welfare, transport, stress, pigs, meat quality

## Abstract

**Simple Summary:**

This study aimed at investigating which pre-slaughter parameters determine variations in the quality of the loin derived from pigs intended for Italian PDO (Protected Designation of Origin) products. Data were collected on 44 commercial shipments of Italian heavy pigs. Meat quality parameters (pH, color lightness, drip loss, cooking loss, and shear force) identified two clusters: ‘Higher Quality’ (HQ) and ‘Lower Quality’ (LQ). The parameters which differed more widely between the two clusters were journey duration, ambient temperature, distance traveled and irregular behaviors (slipping, falling, and overlapping) at unloading. Among the pre slaughter parameters which negatively affect pork quality, consideration should be given to ambient temperatures above 22 °C, distance traveled above 26 km, travel duration between 38 and 66 min, and more than 5.9% of animals showing irregular behaviors at unloading. Journeys involving one or more of these risk factors may require additional attention in terms of animal welfare in order to obtain meat suitable for high-quality productions.

**Abstract:**

This study focused on loin quality in Italian heavy pigs intended for the production of PDOs (Protected Designation of Origin) products, and investigated the pre-slaughter factors which negatively affect the quality of fresh meat. Data were collected on 44 shipments (loads) of pigs. Shipments were carried out under commercial conditions. Several pre-slaughter parameters were recorded within the entire process (on-farm, during transport, and at the slaughterhouse). On a subset of pigs (10 animals from every load, N = 440), serum cortisol and creatine kinase were measured and loin samples were analyzed for pH, instrumental color, drip loss, cooking loss, shear force, and sensory quality. Cluster analysis of the instrumentally-assessed meat quality parameters allowed the categorization of the shipments into two clusters: lower quality (LQ) and higher quality (HQ). Our results showed that the factors with significant differences between the two clusters were journey duration, ambient temperature, distance traveled, and irregular behaviors (slipping, falling, and overlapping) at unloading (all greater in LQ, *p* < 0.05). The pre-slaughter conditions associated with lower loin quality were ambient temperatures above 22 °C, distance traveled above 26 km, travel duration between 38–66 min, more than 5.9% of animals showing irregular behaviors at unloading.

## 1. Introduction

Animal welfare and meat quality in relation to road transportation have received much attention in terms of experimental research, but the effects of road transportation on animal welfare are difficult to monitor and validate under practical conditions, because of the numerous situations and variables which can change during the process (e.g., road condition, driver experience, animal handling, microclimate, etc.) [1]. Besides, according to a recent review [2] the literature currently available on pig transportation mostly focuses on pigs weighing between 100 and 135 kg, and little information is available on heavier pigs, which may react differently to transportation stressors. Besides the case of Italy, where pigs are traditionally transported and slaughtered at a minimum age of 9 months and at the average body weight (BW) of 160 kg ± 10% for the production of typical PDO (Protected Designation of Origin) products (such as Parma Ham, Salame Brianza, Coppa Piacentina, etc.) [3], other European and worldwide countries have been increasing pigs’ slaughtering BW over the past decade. This increase is driven by both the dilution of fixed production cost over more weight per pig and the improvement of genetic selection of lean-type pigs [4]. Several studies evaluated the effects of greater slaughter weights on profitability, carcass quality, primal cuts yield, and pork quality (e.g., [5,6,7,8,9,10]); however, only in a few studies the slaughter weight considerably exceeded 125 kg [5,10]. This framework results in a pronounced lack of knowledge concerning the quality of the fresh meat of heavy pigs, and in particular on how pork quality of this productive category may be affected by pre-slaughter stressors under conventional conditions. Additionally, according to a recent review, the quality of dry-cured hams, which are the most valuable products, greatly depends on raw material properties (e.g., meat physicochemical properties such as pH and water holding capacity) and intrinsic properties of the muscle (moisture, fat, and enzyme activities), all of which are influenced by various ante-mortem factors [11]. The few available studies on pre-slaughter handling of Italian heavy pigs highlighted that animal losses increase when animals are kept in lairage overnight at the plant [12]. This effect, however, was observed to vary largely, depending on the characteristics and management of the lairage area at the slaughterhouse (i.e., presence of large open windows, stocking density in the lairage pens, and the use of cooling devices such as sprinklers) [13]. When heavy pigs are subjected to pre-slaughter handling and short transportation, the parameters found to have a negative impact on blood stress indicators were the following: high average speed of the vehicle during transport, low welfare index at slaughter, low TSWI (overall transport and slaughter welfare index), greater distance traveled, and greater percentage of irregular behaviors (slips, falls, and overlaps) during unloading [14]. However, in the mentioned studies, the consequences of different pre-slaughter handling on meat quality have not been evaluated [12,13], or have produced results inconsistent with the level of stress experienced by the animals (evaluated in terms of blood indicators) [14].

The aim of the present paper, which is part of a wider research project [14], was to fill this lack of knowledge by investigating the relationships between a some pre-slaughtering parameters (measured upon departure, during transport, and at slaughter) and loin quality, to identify which pre-slaughtering have the largest impact on pork quality. Within the framework of a typical pork production mainly focused on cured products, the identification and control of the parameters that have a positive effect on meat cuts to be consumed fresh can help reduce food wastage and promote higher standards of animal welfare.

## 2. Materials and Methods

### 2.1. Ethical Statement

The experiment did not include any invasive procedure in vivo, therefore no specific authorization by the institutional ethical committee was required (Directive 2010/63/EU [15]). Rearing, transport, and slaughtering were carried out in commercial farms/trucks/plants and according to the EU legislation (Directive 2008/120/EC [16], Council Regulation-EC-no. 1/2005 [17], and Council Regulation-EC-no. 1099/2009 [18]).

### 2.2. Experimental Design and Data Collection

Data were collected by monitoring 44 shipments of Italian heavy pigs. As per common commercial practice, animals were raised and transported in mixed-sex groups (gilts and barrows). Shipments originated from 11 randomly-selected commercial farms located in Northern Italy (Emilia Romagna region), with each farm providing 4 loads of pigs (2 during the warm and 2 during the cold season). All animals involved in the study were commercial hybrids suitable for the production of Parma hams according to the production rules [3]. Loads varied between a minimum of 59 and a maximum of 145 pigs (average: 118). Descriptive statistics of the transports analyzed in the present study can be found in Sardi et al. [14]. All animals were transported to the same commercial slaughter plant, where they were electrically stunned and then immediately exsanguinated. For each of the shipments, the experimental protocol included: (a) the assessment of several pre-slaughtered parameters; (b) the collection of blood samples from a subset of pigs, for the evaluation of stress biomarkers; and (c) the collection of meat samples from the same subset of animals, for meat quality and sensory evaluations. The methods by which these data/samples were collected and analyzed are fully described in a previous paper [14] and will be briefly outlined in this section.

#### 2.2.1. Pre-Slaughter Parameters Checklists and Scores/Indexes Calculation

For every shipment (or load) of pigs (N = 44), several pre-slaughter parameters were assessed by filling three checklists: one upon departure from the farm, one during transport, and one at slaughter. All observations were carried out by the same two trained assessors, who worked together. Data were then submitted to a calculation system which allowed the calculation of (1) a farm (on departure) score; (2) a transport score; (3) a slaughter score, and (4) an overall TSWI (Transport and Slaughter Welfare Index). Additional information on the assessment method, together with a full list of the parameters assessed and how they were scored can be found in Barbari et al. [19] and in Sardi et al. [14]. Briefly, the characteristics of the indexes/scores can be summarized as follows:

- On departure (from the farm) checklist. The parameters assessed were: loading duration, path from the pen to the truck (length, width, design, flooring, presence of internal and external corridors, ramps, loading facilities), time taken to move the pigs, handling (tools used and mode of use), irregular behaviors during handling (pigs slipping, falling, overlapping). The integration of observations made according to this checklist resulted in an ‘on-departure score’, whose values could range from the lowest theoretical value of −30.5 points (pts) for the minimum possible welfare level to the highest theoretical value of 15.5 pts for the maximum possible welfare level.
- Transport checklist. The parameters assessed were: distance and duration of the journey, space allowance per pig, presence and number of drinkers, cooling systems, other characteristics of the lorry (possibility to inspect animals and take care of them, internal illumination, floor type and condition, presence or absence of bedding). The integration of the observations made according to this checklist resulted in a ‘transport score’ whose values could range from the lowest theoretical value of −18.0 pts for the minimum possible welfare level to the highest value of 9.5 pts for the maximum possible welfare level.
- Slaughter checklist. The parameters assessed were: unloading duration, path from the truck to the lairage pen and from the lairage pen to the stunning area (flooring, passages, presence of one-way gates), handling (tools used and mode of use), irregular behaviors during handling (pigs slipping, falling, overlapping), lairage pens characteristics (stocking density, ventilation, illumination, thermal insulation, conditions of floors and surfaces, type of pens, presence of mobile partitions, drinkers, cooling systems), stunning area characteristics (partitions, gates, devices, stunning method, stun-to-stick interval, procedures for the use and to check the efficiency of the stunning system, emergency stunning procedures, training of the personnel involved). The integration of the observations made according to this checklist resulted in a ‘slaughter score’, which could range from the lowest theoretical value of −41.5 pts for the minimum possible welfare level to the highest theoretical value of 50.5 pts for the maximum possible welfare level.
- TSWI: The Transport and Slaughter Welfare Index summarizes the welfare experienced by the animals during the entire pre-slaughter period (from when they are taken from their pen at the farm until when they are slaughtered). The TSWI integrated the three previously calculated scores and its theoretical values could range from a minimum of −90.0 pts for the lowest possible welfare level to a maximum of 75.5 for the highest possible welfare level.

#### 2.2.2. Blood Sampling and Analysis

Blood samples were collected at exsanguination from 10 randomly-selected animals from each of the 44 loads (N = 440). Blood was refrigerated and transferred to the laboratory of the Department of Veterinary Medicine of the University of Bologna (Italy), where plasma was immediately separated and stored at −20 °C, pending subsequent analysis for cortisol, creatine kinase, and aldolase. Cortisol was used in this study as an indicator of acute pre-slaughter stress (e.g., during handling, transport, and/or restraint) [20]. Creatine kinase (CK) was chosen as a subacute indicator of intense physical activity (due for example to exhaustion, loading, or harsh handling) and as a biomarker of overall welfare during transport [21]. Blood CK concentration rises up to a peak approximately 6 h after muscle damage and returns to basal levels between 8 h and 48 h [22,23]. Aldolase is a less-studied, possible long-term indicator of muscle damage. Its blood concentration peaks after at least 48–72 h from the damage [24] and its activity has been correlated to meat quality traits such as pH and myofibrillar protein solubility [25]. Aldolase has been used in human medicine as a more accurate and objective indicator of muscle damage than CK due to its lower inter-individual variability [24]. In the present paper, cortisol concentration (which will be expressed as ng/mL) was measured using a radioimmunological assay technique [26], whereas creatine kinase and aldolase concentrations in plasma (which will both be expressed as U/L) were measured using two commercially available kits based on a colorimetric method with subsequent spectrophotometric UV readings (CK Nac Liquid and Aldolase, Sentinel Diagnostics, Milano, Italy).

#### 2.2.3. Meat Sampling and Meat Quality Assessment

The meat parameters were measured on the longissimus thoracis and lumborum (LTL) muscle of the same animals from which the blood samples were collected (10 animals per shipment, N = 440). At the slaughter plant, carcass lean content was measured using a Fat-o-Meater (F-o-M SFK, Copenhagen, DK), and pH at 45 min (pH 45) was measured in the LTL muscle (at the second/third last rib level) using a portable pH meter equipped with a glass spear tip electrode and a temperature compensation probe (model 250A—Orion Research, Boston, MA, USA). Two portions of the LTL muscle were collected from each carcass, refrigerated and transferred to the laboratory for the assessment of instrumental color, pH at 24 h post mortem (pH 24), Drip Loss, Cooking Loss, and Warner–Bratzler Shear Force (WBSF). Measurements of pH were carried out by means of the same portable pH meter described above. Color was measured according to the CIE Lab (lightness index L*, green/red index a*, blue/yellow index b*) color space system [27] using a Minolta Chromameter set with D65 illuminant (model CR-400—Konica Minolta optics INC., Tokyo, Japan). Drip Loss, Cooking Loss (water bath cooking until core temperature reached 75 °C), and WBSF were assessed subsequently on the same meat sample according to the methods described previously by Honikel [28] and also reported by Sardi et al. [14].

The second muscle portion was transferred to the laboratories at the Research Centre on Animal Production (Reggio Emilia, Italy). Here samples were vacuum-packed and stored at −20 °C pending sensory analysis, which was carried out within a month by a panel of trained experts, according to the method described by Della Casa et al. [29]. The sensory analysis included a visual assessment on raw samples (lean color and marbling) and a subsequent evaluation of the cooked sample (initial tenderness, chewing tenderness, juiciness, final residue, chewiness, aroma intensity, buttery aroma, and off-flavors). Photographic scales were provided for the assessment of lean color (0 = light pink, reference sample chicken breast; 10 = brown, reference samples horse steak after a few days) and marbling (0 = absent or low, on one side of the slice; 10 = high on all sides of the slice) [29]. All other factors were evaluated using a structured continuous scale with values ranging between 1 and 10 (1 = sensation absent, 10 = sensation of the greatest intensity).

### 2.3. Statistical Analysis

Statistical analysis was carried out using the software JMP v15.1 (SAS Institute Inc., Cary, NC, USA). Meat quality parameters (pH45, pH24, L*, Drip Loss, Cooking loss and WBSF) within the corresponding shipment were used in a k-means cluster analysis to differentiate clusters of animals based on their instrumentally-assessed meat quality. Three different models (with two, three, and four clusters) were tested and compared for their cubic clustering criterion (CCC) values. The two-cluster model was chosen, based on its greater fit statistics (CCC values of −1.4887, −3.1458, and −4.7393 for the two-, three-, and four-cluster model, respectively). The two-cluster model allowed the identification of two groups, including 34 and 10 shipments (clusters characteristics will be described more in detail in Section 3.1). For numerical variables, a linear mixed-model procedure was used to identify statistical differences between the two clusters in the pre-slaughter measures (including measures taken on departure, during transport, and at slaughter). Each shipment from the farm of origin was considered as an experimental unit and used as a random variable for all analyses. For blood parameters and meat quality, each shipment was used as the experimental unit, and the 10 measures for each shipment were considered as repeated measures with an autoregressive covariance structure (AR1). The cluster grouping was used as a fixed effect within the model. Means were separated based on least-square mean, and all pairwise multiple comparisons were carried out using Tukey post-hoc test. For categorical variables, a nominal logistic model with the chi-square likelihood ratio test was carried out. A *p*-value ≤ 0.10 was considered as a tendency, a *p* ≤ 0.05 was considered statistically significant.

A random K-fold (5-fold) neural network model was also run to recognize underlying relationships between the meat quality clusters and the pre-slaughter variables. In the training set, the R-square statistic and the RMSE (root-mean-square error) were 0.9307 and 0.1469, respectively. In the validation set, the R-square statistics and the RMSE were 0.9032 and 0.1703, respectively. A ROC (receiver operating characteristic) curve procedure was used to identify thresholds, sensitivity, and specificity of a list of individual pre-slaughter variables.

## 3. Results and Discussion

### 3.1. Clusters Based on Meat Parameters

**Figure 1:**
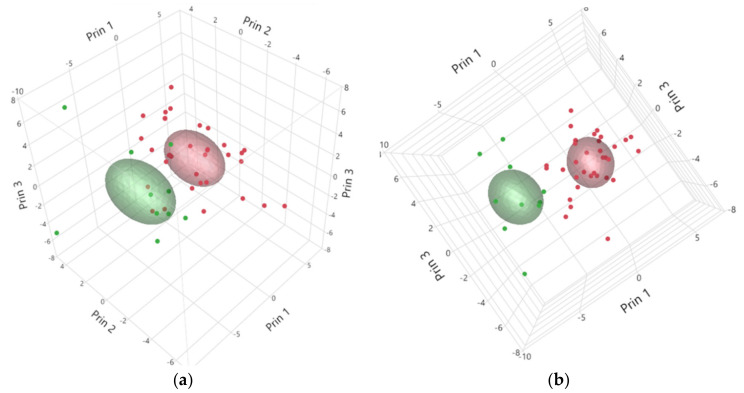
Three-dimensional representation of the shipments clustered according to meat quality parameters (pH 45, pH 24, L*, Drip Loss, Cooking Loss and WBSF) and seen from different angles (**a**) and (**b**). Each dot on the graph represents a shipment. Cluster 1 (red color, 34 shipments), Cluster 2 (green color, 10 shipments). The colored area represents the area around the cluster centroid.

Meat quality parameters (pH45, pH24, L*, Drip Loss, Cooking Loss and WBSF) allowed the separation of the shipments in two clusters (which are graphically shown in Figure 1). Cluster 1 (red color) included 34 shipments (N = 340 meat samples), and cluster 2 (green color) included 10 shipments (N = 100 meat samples). The average value of the main instrumental meat quality parameters for the two clusters and the statistical differences between clusters are summarized in Table 1.

Concerning the parameters used for clusterization, Cluster 1 included shipments with animals having significantly greater (*p* < 0.0001) average pH45′ and pH24 h, as well as significantly lower (*p* < 0.0001) L* and Drip Loss values than Cluster 2. Cooking Loss tended to be lower (*p* = 0.064) in Cluster 1 than in Cluster 2. For these reasons, Cluster 1 and Cluster 2 were renamed Higher Meat Quality (HQ), and Lower Meat Quality (LQ), respectively. Shear force (WBSF) was significantly greater in HQ than in LQ cluster (*p* < 0.0001). Significant differences were observed also in a*, b*, and Chroma values, which were all greater in Cluster 2 (LQ). Carcass lean content was similar between clusters.

Overall, the meat quality profile of Cluster 1 (HQ) indicates more favorable characteristics, due to the more limited rate and extent of pH decline, leading to a lower degree of protein denaturation and disintegration of cellular structures [30], resulting also in greater water holding capacity (WHC) both during conservation at +4 °C (drip loss) and during cooking (cooking loss). The combination of a high protein denaturation degree and a low WHC usually results in light meat color, due to greater light-scattering properties of the shrunk proteins, as it also happens in meat with the PSE (pale, soft, and exudative) defect [31]. Accordingly, greater L* values were observed in Cluster 2 (LQ), compared to Cluster 1 (HQ). The main characteristics of the LQ meat are compatible with the PSE definition, including also the consistency of meat assessed on the cooked samples, which was softer (lower WBSF values) in the LQ than in the HQ cluster. While several studies observed a reduced tenderness of PSE meat [32], our results seem to agree with Tornberg, who hypothesized that, when meat is cooked at temperatures above 60 °C, the WBSF decreases as the degree of contraction of the fibers increases [33]. This would imply that cooking PSE meat, which has a greater degree of longitudinal contraction, would result in a greater tenderness (i.e., a lower WBSF). It is, however, noteworthy that the drip loss observed in heavy pigs meat (being it either normal or similar to PSE) is usually considerably lower than what observed in lighter animals (e.g., [34,35]), probably due to the different age and weight at slaughter, reflecting on the moisture content of the carcass (and meat) [36].

Statistically significant differences between clusters were observed also with respect to the instrumentally-assessed meat color, with LQ cluster having greater redness index (a* value) and yellowness index (b* value), and an overall greater chroma value than HQ (*p* < 0.05 for the three variables). Such small differences in color, likely not detectable by the human eye, probably indicate that the meat belonging to the LQ cluster may fall within the case of untypical deviations like RSE (reddish-pink, soft, exudative), which can be defined as a mild occurrence of PSE [30,35].

### 3.2. Differences in Transport Variables, Blood Parameters, and Sensory Meat Quality between Clusters

Table 2 shows the linear mixed model applied to some of the pre-slaughter variables measured in the TSWI checklists.

According to our model, some of the pre-slaughter parameters statistically differed between the two clusters. In particular, the variables journey duration, ambient temperature, distance traveled, and irregular behaviors (total of slipping, falling, and overlapping) observed at unloading were all significantly greater (*p* < 0.05) in the LQ than in the HQ cluster. The other variables and the calculated scores/indexes (on-departure score, transport score, slaughter score, and TSWI) did not significantly differ between clusters.

Despite the statistically significant differences found between the two clusters, it can be observed that some variables, such as transport duration and distance traveled, indicate that on average both groups of animals were transported for a short time and over small distances (for journey duration: average 37.2, SD 18.8, minimum 18, maximum 90 min; for distance traveled: average 26.2, SD 11.6, minimum 11, maximum 59 km, data shown in [14]). Short travel duration is common for Italian heavy pigs, and previous research indicated that between one half and 90% of these animals are transported for less than 2 h [12,13]. In the present study, however, differences in meat quality were observed despite the short average transportation time, with relatively greater transportation times and greater distances traveled negatively affecting meat quality. This result is in partial disagreement with the findings on lighter pigs of Warriss et al. [37], who compared two relatively short travel durations (1 h vs. 4 h) and found no major effects on meat quality. This may indicate that the meat quality of heavy pigs is more subjected to alterations even during short time transportations. It is, however, worth observing that, under some circumstances, short time (1 h) transportation has been deemed to be more stressful than longer ones (3 h) because, if conditions on the truck are good, during the journey animals can recover from the stress suffered at loading [38]. In our case, this may indicate that the transport conditions were quite good regardless of the cluster, and this is confirmed by the transport score value, which was similar between the clusters (3.10 vs. 3.90) and fell in the moderate-to-high welfare range. In fact, as shown in the Materials and Methods section, transport score ranges from −18 pts for the lowest welfare level possible to 9.5 pts for the highest welfare level; therefore, values of about 3–4 pts collocate the transport in the top 25% of the assessment checklist, indicating that most of the required parameters illustrated in Sardi et al. ([14]) were met.

The differences observed concerning ambient temperature are in agreement with the literature. It is well known that acute heat stress before slaughter stimulates muscle glycogenolysis and can therefore lead to low WHC and to PSE meat [39]. Accordingly, a study on lighter pigs (127 kg BW) showed that transports carried out during summer resulted in paler meat color (i.e., in greater L* value) than during winter [40]. Čobanović et al. [34] observed the lowest pH and the highest thawing loss, L* and b* values, and occurrence of PSE meat in pigs (110 kg BW) transported during summer.

To the best of our knowledge, no study specifically investigated the relationship between irregular behaviors and meat quality, although it has been observed that harsh handling or bad facility design (resulting in more slips, falls, and overlaps), especially at loading, is correlated with more intense stress response and reduced pork quality [41,42]. This may warrant the need for future studies on behavior during unloading.

Table 3 shows the results of the linear mixed model applied to the blood parameters and to the sensory meat quality traits. Our results show no substantial difference between clusters in stress and muscle damage parameters (cortisol, CK, and aldolase). This seems to confirm the poor-to-moderate relationship between physiological response to pre-slaughter stress and meat quality which has been previously observed in other studies (e.g., [43,44]).

As concerns sensory parameters, from a general standpoint, it can also be observed that several variables, despite not showing statistically significant differences, were in general agreement with the higher vs. lower meat quality characteristics observed in the two clusters (i.e., numerically greater marbling score, initial tenderness, chewing tenderness, juiciness, chewiness, and aroma intensity in the HQ cluster). Conversely, significant differences were observed in some sensory traits, with LQ cluster having a lower (i.e., paler) color score, and a greater final residue and buttery aroma scores. Color and final residue are in agreement with the instrumentally-measured meat characteristics described above (L* and WHC, respectively), whereas the differences in the buttery aroma are harder to explain, especially considering the almost identical marbling score and carcass lean meat content between the two clusters. It should, however, be mentioned that—despite being significant—the difference observed in the buttery aroma was detected by a panel of trained experts and amounted to 0.3 points out of a 10-point sensory scale, therefore it may not be easily perceived by the average consumer. The same observation can also be made for the differences detected in visually assessed color.

### 3.3. Results of the Neural Network Model to Identify Possible Thresholds for Meat Quality Variation in the Pre-Slaughtering Variables

**Figure 2:**
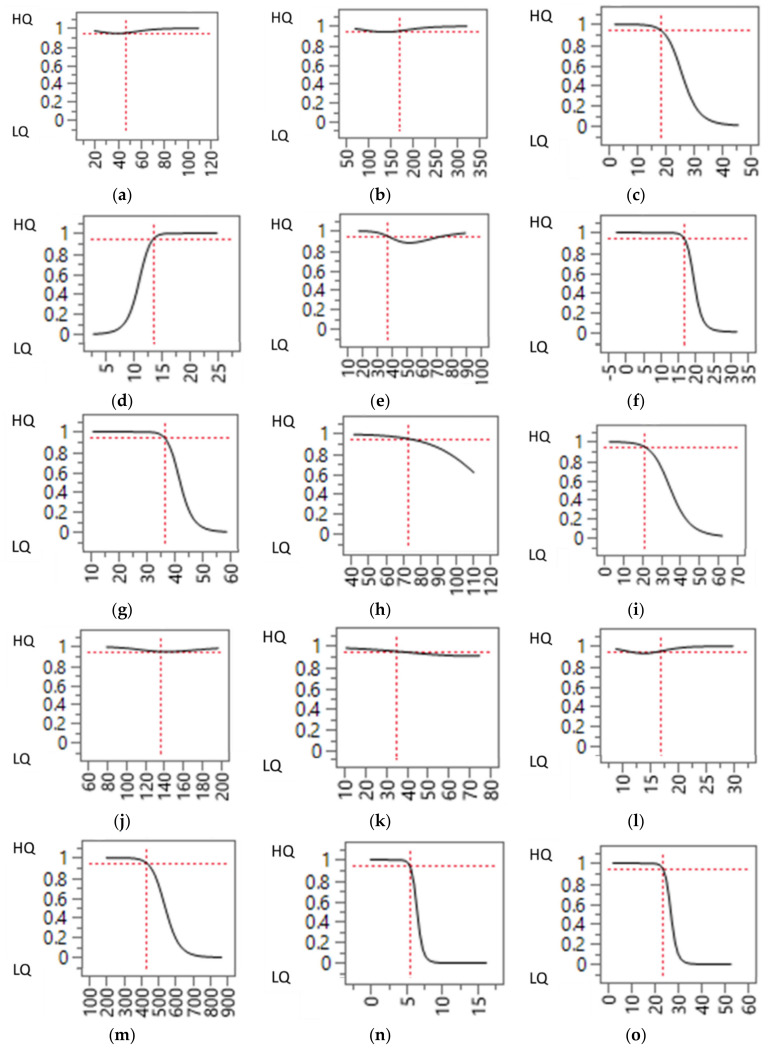

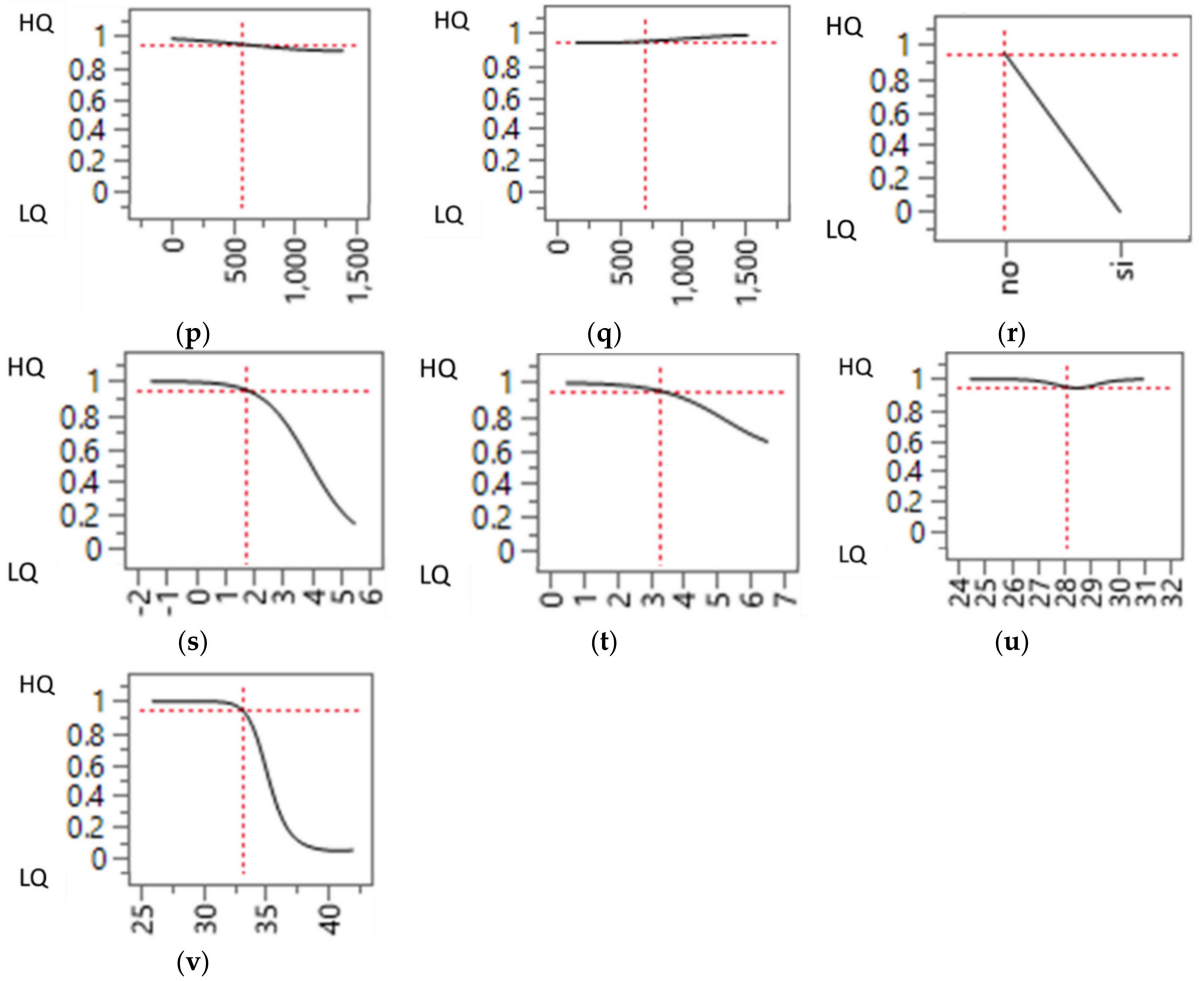
Profile graphs of the neural network model for all the pre-slaughter variables presented in Table 2. For each graph, the name of the variable plotted on the *x*-axis is indicated below the figure. (HQ = High quality cluster; LQ = Low quality cluster). Variable names highlighted in bold character indicate statistically significant differences (*p* < 0.05) between clusters, as presented in Table 2. (**a**) Loading duration, min. (**b**) Pigs loaded per hour, n. (**c**) Irregular behaviors at loading, %. (**d**) Waiting time at the farm, min. (**e**) **Journey duration, min**. (**f**) **Ambient temperature, °C**. (**g**) **Distance traveled, km**. (**h**) Average vehicle speed, km/h. (**i**) Waiting time at slaughterhouse, min. (**j**) Total journey duration, min. (**k**) Total waiting time on the truck, min. (**l**) Unloading duration, min. (**m**) Pigs unloaded per hour, n. (**n**) **Irregular behaviors at unloading, %**. (**o**) Total irregular behaviors. (**p**) Lairage duration, min. (**q**) Transport + lairage duration, min. (**r**) Stable (unmixed) groups, odds ratio. (**s**) On-departure (at farm) score, pts. (**t**) Transport score, pts. (**u**) Slaughter score, pts. (**v**) TSWI, pts.

As concerns the neural network analysis, the high R-Square statistic for the validation set (above 0.9) indicates that the model is predicting well on data not used to train it, and also indicates a good capability of the model to describe a phenomenon. Figure 2 shows the profile graphs resulting from the neural network model for each of the pre-slaughter variables presented in Table 2, to visually understand how the variables affect the predicted meat quality clusterization. For completeness, all variables considered are presented in Figure 2 and briefly described in the next paragraph, but only those significantly differing between the meat quality clusters will be discussed in detail. To improve readability, the caption below each graph indicates the variable plotted on the x-axis. Each graph indicates how the probability of the transport to be classified as either HQ or LQ changes as the pre-slaughter variable indicated on the horizontal axis varies.

This paragraph provides a general description of the results of the neural network shown in Figure 2. Some of the variables (loading duration, number of pigs loaded per hour, total journey duration, total waiting time on the truck, unloading duration, lairage duration, transport + lairage duration, slaughter score) showed no appreciable relation with the cluster classification. Other parameters (i.e., irregular behaviors at loading, average vehicle speed, waiting time at the slaughterhouse, pigs unloaded per hours, total irregular behaviors, groups stability, farm score, transport score, and TSWI) seemed to show an overall negative relationship with the meat quality clusters (as the value of the variables increased, shipments were more likely to be classified in the LQ clusters). However, as previously described, these variables failed to reach statistically significant differences between clusters. Similarly, a positive correlation (leading also in this case to no statistically significant differences between clusters) was observed only for the variable ‘waiting time at the farm’ (as the waiting time increased, the shipments were more likely to be classified in the HQ cluster). This may indicate that a short waiting time at the farm, especially if the weather is mild, may give to the animals the possibility to adapt (at least partially) to the truck environment before the beginning of the journey. According to our statistical model, in fact, a 14-min waiting time increased the probability of having HQ meat (specificity 0.70, sensibility 0.59).

Among the variables which, as described in Section 3.3, statistically differed between the two clusters (journey duration, ambient temperature, distance traveled, irregular behavior at unloading), three (namely ambient temperature, distance traveled, irregular behaviors at unloading) showed a negative correlation with the cluster classification. More specifically, for ambient temperature, the observed cut-off value (as assessed using the ROC curve) was at 22 °C (sensitivity 0.88; specificity 0.60); and transports carried out above this temperature had a greater probability of resulting in lower meat quality. Previous studies on Italian heavy pigs found an increase in transport losses during the summer [12], and in particular when the THI (temperature humidity index) exceeded 78.5 (corresponding, for example, to combinations of temperature and relative humidity of about 35° and 20%, or 30°C and 50%, or 27 °C and 80%) [13]. Our results, therefore, indicate that adverse effects on meat quality can be already observed at lower ambient temperatures. Interestingly, even lower temperatures were indicated in studies on the mortality of market-weight pigs, in which in-transit losses have been reported to increase beyond ambient temperatures of 16–17 °C [45,46]. It is also worth pointing out that, in terms of thermal comfort, a more accurate evaluation of the heat stress level experienced by the animals could be carried out by implementing more complex measurements, such as continuous monitoring of the pigs’ internal temperature [47], monitoring the microclimate inside the tuck (e.g., by assessing times derivatives of temperature or enthalpy [48]) or including a quantification of the heat and moisture generated by animals during transportation [49]. Such complex evaluations, however, fall outside the scope of the present paper, in which the evaluation of pre-slaughter variables was carried out only using a checklist system and no sensors were installed on the truck or placed on/inside the animals’ body.

As concerns irregular behaviors at unloading, the observed threshold was at 5.9% (sensitivity 0.74; specificity 0.90), implying that transports in which more than this percentage of animals showed slipping, falling, or and overlapping at unloading were more likely to belong to the LQ cluster.

As concerns distance traveled, the cut-off value was 26 km (sensitivity 0.80; specificity 0.79), with animals transported for 26 km or above having lower meat quality. Journey duration showed a bimodal trend, in which transports lasting between 38 min (sensitivity 0.80; specificity 0.73) and 66 min (sensitivity 0.30; specificity 0.94) were associated with worse meat quality. This seems to indicate that shorter transports do not consistently affect meat quality. As concerns longer journeys, it has previously been observed that, provided that conditions on the means of transportation guarantee adequate comfort, animals may (at least partially) recover from the stress and muscular fatigue experienced during loading [38].

These threshold values could be used as indicators (or risk factors) to identify which shipments of heavy pigs deserve particular attention. Guaranteeing better transportation conditions during these shipments (e.g., by installing cooling systems on the trucks, reducing stocking densities, providing adequate bedding, etc.) can help to improve meat quality, together with animal welfare. However, given the relatively limited variability of some of the examined parameters (e.g., distance traveled, transportation scores, journey duration), these results should be validated under more variable transport conditions. Additionally, it should also be observed that the calculated welfare indexes (on-departure score, transport score, slaughter score, and TSWI) showed no appreciable correlation with meat quality, probably because all the transports monitored in this study fell in the moderate-to-high animal welfare level, reducing the variability in the calculated indexes [14]. For all the above reasons, further validation of these results under more variable pre-slaughter welfare conditions is warranted.

## 4. Conclusions and Future Work

This study aimed at identifying the pre-slaughter parameters which cause the largest variation in the meat quality of Italian heavy pigs. A wide range of pre-slaughter variables was assessed, alongside with some previously-described indexes and scores (on-departure score, transport score, slaughter score, and TSWI). Our results identified some pre-slaughter conditions (ambient temperatures above 22 °C, distance traveled 26 km or above, travel duration between 38 and 66 min, more than 5.9% of animals showing irregular behaviors at unloading) which increased the risk of worse meat quality in Italian heavy pigs. While some of the described effects have previously been observed (despite at different thresholds) also in lighter pigs, to the best of our knowledge, no study addressed the relationship between irregular behaviors during unloading and meat quality. Notwithstanding the need for further validation of our results, these parameters offer a first guide for the identification of those shipments that may require additional attention in terms of animal welfare in order to obtain meat suitable for high-quality productions. Lastly, additional ethical consideration should be drawn to the fact that, despite transports were carried out under overall moderate-to-high welfare conditions, as much as 25% of the loins analyzed in the present study were classified as ‘lower quality’. This aspect urges a wider reflection on possible strategies to be adopted in order to improve animal welfare and product quality.

## Figures and Tables

**Table 1:** Summary of the meat quality characteristics of the two clusters. Variables in italics were used for data clusterization.

Parameter, U.M. ^3^	Cluster 1 (HQ ^1^) (N = 340 Pigs)		Cluster 2 (LQ ^2^) (N = 100 Pigs)		*p*-Value
	Estimate	SE ^4^	Estimate	SE ^4^	-
*pH 45*	6.06	0.02	5.80	0.04	<0.0001
*pH 24*	5.54	0.006	5.43	0.01	<0.0001
L*	46.6	0.5	53.0	0.9	<0.0001
*Drip Loss, %*	0.98	0.04	1.47	0.08	<0.0001
*Cooking loss, %*	25,8	0.6	28.1	1.0	0.0643
*WBSF* ^5^ *, kg/cm^2^*	4.0	0.1	3.4	0.2	0.0054
F-o-M ^6^	48.6	0.2	48.3	0.4	0.3413
a*	3.9	0.2	4.7	0.3	0.0491
b*	4.6	0.2	5.9	0.3	0.0002
Hue ^7^	0.89	0.01	0.91	0.02	0.382
Chroma ^8^	6.2	0.2	7.7	0.4	0.0016

^1^ Higher Quality; ^2^ Lower Quality; ^3^ Unit of Measurement; ^4^ Standard Error; ^5^ Warner–Bratzler Shear Force; ^6^ Carcass lean content was measured using a Fat-o-Meater; ^7^ Hue = √(a*^2^ + b*^2^); ^8^ Chroma = arctan(b*/a*).

**Table 2:** Results of the linear mixed-model procedure to identify statistical differences between the two clusters in the measured transport and slaughter variables.

Number of Shipments	HQ ^1^		LQ ^2^		
	34		10		
Variable	Estimate	SE ^3^	Estimate	SE	*p*-Value
Loading duration, min	47.7	3.7	43.9	6.9	0.6469
Pigs loaded per hour, n	170	11	170	20	0.9870
Irregular behaviors (slipping, falling, overlapping) at loading, %	17.3	1.9	21.0	3.4	0.3362
Waiting time at the farm (before departure), min	14.3	0.8	11.5	1.5	0.1173
Journey duration, min	33.8	3.1	48.6	5.7	0.0277
Ambient temperature, °C	15.3	1.4	21.9	2.6	0.0291
Distance traveled, km	23.3	1.8	36.1	3.3	0.0014
Average vehicle speed during transport, km/h	72.0	2.8	77.7	5.2	0.3433
Waiting time at the slaughterhouse (before unloading), min	20.4	2.5	24.2	4.7	0.4796
Total journey duration (from loading to unloading), min	133.4	5.6	143.7	10.4	0.3906
Total waiting time on the truck (farm + slaughterhouse), min	34.7	2.8	35.7	5.2	0.8672
Unloading duration, min	17.4	0.9	15.5	1.6	0.3209
Pigs unloaded per hour, n	427	22	452	40	0.5919
Irregular behaviors (slipping, falling, overlapping) at unloading, %	4.82	0.58	7.77	1.06	0.0192
Total irregular behaviors (slipping, falling, overlapping), %	22.1	2.1	28.8	3.8	0.1243
Lairage duration (from unloading to stunning), min	576	89	528	164	0.7986
Transport + lairage duration, min	710	90	672	165	0.8423
Stable (unmixed) groups, odds ratio	0.59	0.39	0.70	0.39	0.5183
On-departure (at farm) score, pts	1.66	0.29	2.05	0.55	0.5391
Transport score, pts	3.10	0.28	3.90	0.51	0.1798
Slaughter score, pts	27.97	0.39	28.45	0.73	0.5645
TSWI ^4^ (farm + transport + slaughter), pts	32.73	0.59	34.40	1.09	0.1875

^1^ Higher quality, ^2^ Lower quality; ^3^ Standard error; ^4^ Transport and Slaughter Welfare Index.

**Table 3:** Results of the linear mixed-model procedure to identify statistical differences between the two clusters in the measured blood parameters and sensory attributes of meat. Back-transformed data are presented between square brackets.

Number of Samples	HQ ^1^		LQ ^2^		
	340		100		
Variable	Estimate	SE ^3^	Estimate	SE	*p*-Value
log Cortisol, ng/mL	1.033 [10.79]	0.030	1.040 [10.96]	0.054	0.9176
log CK ^4^, U/L	3.291 [1954]	0.01 4	3.263 [1832]	0.026	0.3623
log Aldolase, U/L	1.647 [44.36]	0.027	1.618 [41.50]	0.049	0.605
Color score	4.72	0.06	4.45	0.09	0.0126
Marbling score	4.96	0.11	4.89	0.17	0.7300
Initial tenderness	5.52	0.11	5.27	0.18	0.2676
Chewing tenderness	5.07	0.11	4.80	0.18	0.2282
Juiciness	3.88	0.13	3.76	0.21	0.6127
Final residue	2.90	0.05	3.16	0.07	0.0076
Chewiness	5.43	0.09	5.31	0.14	0.4788
Aroma intensity	5.38	0.12	5.27	0.18	0.5929
Buttery aroma	2.81	0.06	3.08	0.10	0.0318
Off-flavors	2.28	0.05	2.31	0.08	0.7858

^1^ Higher quality, ^2^ Lower quality; ^3^ Standard error ^4^ Creatine kinase.
